# Genetic Structure of Cucumber Mosaic Virus From Natural Hosts in Nigeria Reveals High Diversity and Occurrence of Putative Novel Recombinant Strains

**DOI:** 10.3389/fmicb.2022.753054

**Published:** 2022-02-10

**Authors:** Oluropo A. Apalowo, Adedapo O. Adediji, Olusegun S. Balogun, Temitope I. Fakolujo, Joy M. Archibong, Nkechi B. Izuogu, Mohamed A. Abdelgawad, Mohammed M. Ghoneim, Suleiman Mustapha, Fadi S. I. Qashqari, Gaber E. Batiha, Gabriel I. Atiri

**Affiliations:** ^1^Department of Crop Science and Horticulture, Faculty of Agriculture, Nnamdi Azikiwe University, Awka, Nigeria; ^2^Department of Crop Protection, Faculty of Agriculture, University of Ilorin, Ilorin, Nigeria; ^3^Department of Crop Protection and Environmental Biology, Faculty of Agriculture, University of Ibadan, Ibadan, Nigeria; ^4^Department of Pharmaceutical Chemistry, College of Pharmacy, Jouf University, Sakaka, Saudi Arabia; ^5^Department of Pharmacy Practice, Faculty of Pharmacy, AlMaarefa University, Ad Diriyah, Saudi Arabia; ^6^Division of Crop Protection, ICAR-Indian Institute of Horticultural Research, Bengaluru, India; ^7^Department of Microbiology, College of Medicine, Umm Al-Qura University, Makkah, Saudi Arabia; ^8^Department of Pharmacology and Therapeutics, Faculty of Veterinary Medicine, Damanhour University, Damanhour, Egypt

**Keywords:** pepper (*Capsicum annuum* L.), tomato (*Solanum lycopersicum* L.), African garden eggplant (*Solanum aethiopicum* L.), watermelon (*Citrullus lanatus* Thumb.), subgroup IB, phylogeny, recombination, negative selection pressure

## Abstract

Cucumber mosaic virus (CMV, *Bromoviridae*: *Cucummovirus*), one of the most widespread plant viruses with several hosts, causes huge losses in yield quality and quantity. The occurrence of various CMV strains and high genetic diversity within the virus complicate its management. We describe the population structure of CMV in Nigeria using partial RNA1 and RNA3 gene sequences from three natural hosts: pepper (*Capsicum annuum*), tomato (*Solanum lycopersicum*), and watermelon (*Citrullus lanatus*). One hundred and six leaf samples were obtained from 16 locations across Nigeria, and specific primers were used to amplify the two gene fragments using PCR. Twenty-four samples tested positive for CMV using RNA1 primers, and amplicons were sequenced from 12 isolates, revealing 82.94–99.80% nucleotide and 85.42–100% amino acid sequence similarities within the population. The partial RNA3 fragment, corresponding to the complete coat protein (CP) gene, was sequenced from seven isolates, with 95.79–97.90% and 98.62–100% nucleotide and amino acid intrapopulation similarities, respectively. The isolates belonged to subgroup IB and formed distinct phylogenetic clusters in both gene sets, indicating putative novel strains. Recombination signals, supported by phylogenetic inferences, were detected within the RNA1 dataset (*P* ≤ 0.05) and identified a recombinant isolate within the Nigerian sequences. No recombination was detected within the CP genes. Population genetics parameters established high diversity within the Nigerian population compared to other isolates worldwide, while selection pressure estimates revealed the existence of negative selection in both gene sets. Although CMV subgroup IB strains were postulated to originate from Asia, this study reveals their prevalence across several hosts from different locations in Nigeria. To our knowledge, this is the first comprehensive description of a recombinant CMV subgroup IB isolate from West Africa, which has implications for its robust detection and overall management.

## Introduction

Cucumber mosaic virus (*Bromoviridae*: *Cucumovirus*) is a cosmopolitan virus that is considered to be economically important because of its ability to infect thousands of hosts worldwide (Zitter and Murphy, 2009; Jacquemond, 2012). The virus is widespread and can be found across temperate and tropical climates, affecting many agricultural and horticultural crops (Rossinck et al., 1999). It consists of icosahedral particles of 29 nm diameter and 180 capsid protein subunits (Palukaitis and García-Arenal, 2003), while its molecular weight falls in the range of 5.8 to 6.7 million, consisting of approximately 18% RNA (Francki and Hatta, 1980). The virus genome consists of three messenger sense, single stranded RNAs, designated 1, 2, and 3, in order of decreasing size (Palukaitis et al., 1992). RNAs 1 and 2 are encapsulated separately (Hayes and Buck, 1990), whereas RNA-3 and subgenomic RNA-4 are encapsidated within the same particle (Canto et al., 1997). These segments produce the virus 1a (replicase), 2a, 2b, 3a (movement), and 3b (capsid) proteins (Bujarski et al., 2012). Some CMV strains support a satellite RNA, designated RNA 5 or satRNA, a single-stranded molecule of approximately 332 to 342 nucleotides that is completely dependent on CMV for its replication (Gonsalves et al., 1982). Moreover, satRNA is encapsidated in CMV particles, which allows spread along with CMV and by its aphid vectors (Zitter and Murphy, 2009). The virus has the widest host range among all known plant viruses, attacking a great variety of vegetables, ornamentals, and other plants comprising more than 1,500 host species across 40 families (Palukaitis et al., 1992).

Numerous CMV strains have been classified into two major subgroups, I and II, on the basis of serological properties and nucleotide sequence homology (Palukaitis et al., 1992; Roossinck, 2001). Subgroup I was further divided into two subgroups (IA and IB) based on phylogenetic analysis and the 3′UTR of RNA-3 (Rossinck et al., 1999). These numerous strains differ in their hosts, symptom expression, mode of transmission and characteristics (Jacquemond, 2012). Strains belonging to subgroups IA and II have worldwide occurrence, while subgroup IB has Asian origins (Roossinck, 2002), although it has been reported across other geographical regions (Aramburu et al., 2007; Farzadfar et al., 2013; Kayode et al., 2014; Mutuku et al., 2018; Kidanemariam et al., 2019). The genetic diversity and strain differentiation in CMV is partly driven by varying degrees of recombination and reassortment (Bonnet et al., 2005; Nouri et al., 2014; Hasiów-Jaroszewska et al., 2017), which is also host-driven (Ouedraogo et al., 2019). This occurs within genomic segments in the same host and similar strains (Pita et al., 2015), across various strains (Chen et al., 2002), and with other cucumoviruses (de Wispelaere et al., 2005). The virus is sap transmissible and can also be transmitted non-persistently by over 80 aphid species (Palukaitis and García-Arenal, 2003). It is also seed transmitted but to a very limited extent.

In Nigeria, CMV has been reported on various hosts, including vegetables and solanaceous crops (Atiri, 1985; Ayo-John and d’A Hughes, 2014; Alegbejo, 2015; Arogundade et al., 2015; Ekpiken et al., 2021). Its molecular and genetic properties in the country have been previously investigated (Kayode et al., 2014; Fakolujo, 2018; Adediji, 2019). However, the characteristics of the virus populations occurring at the molecular level are yet to be fully elucidated. This research attempts to provide information on the genetic characteristics of CMV populations from natural four hosts in Nigeria, pepper (*Capsicum annuum*), tomato (*Solanum lycopersicum*), African garden eggplant (*S. aethiopicum*) and watermelon (*Citrullus lanatus*), using partial RNA1 and complete CP gene fragments. We identified the prevalence of highly divergent CMV isolates belonging to subgroup IB and the occurrence of potential new strains based on phylogenetic inferences. Using these partial genomes, we also detected the presence of a putative recombinant within the virus population with potential implications for virus management in Nigeria.

## Materials and Methods

### Leaf Sample Collections

**FIGURE 1:**
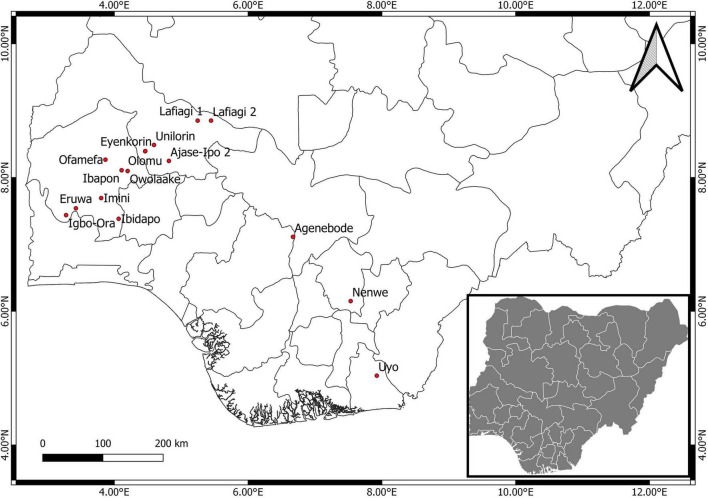
Map of Nigeria showing the areas sampled for CMV symptoms in this study.

**FIGURE 2:**
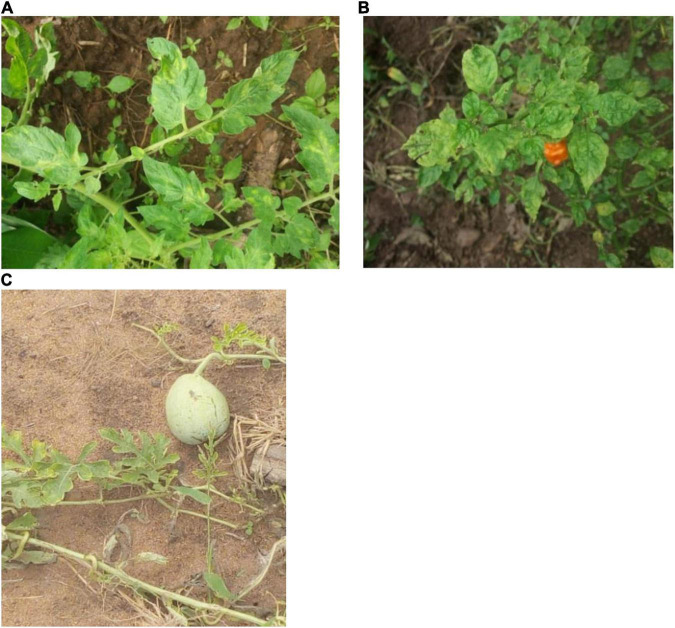
Symptoms on tomato **(A)**, pepper **(B)**, and watermelon **(C)** plants sampled for natural CMV infection in Nigeria.

Leaves showing CMV-like symptoms were obtained from 16 locations across five states (Oyo, Kwara, Edo, Enugu, and Akwa Ibom) in Nigeria (Figure 1). Sampling was performed on four economically important fruit vegetables in Nigeria: pepper, tomato, African garden eggplant, and watermelon. Plants mainly showing chlorosis and mosaic patterns were sampled (Figures 2A–C), while leaves were stored in small plastic vials containing silica gel and cotton wool at ambient temperatures of 25 ± 3°C until laboratory analyses.

### Nucleic Acid Extractions and Reverse Transcription Polymerase Chain Reaction

Total nucleic acids were extracted from the leaf samples using a modified CTAB procedure as previously described by Abarshi et al. (2010). Partial RNA1 and complete CP gene fragments from the CMV genome were amplified from the aqueous extracts using primer pairs CMV-F (5′-CCGCTTGTGCGTTTAATGGCT-3′)/CMV-R (5′-GCCGTAA GCTGGATGGACAA-3′) (Wylie et al., 1993) and CMV-CP-F (5′-ATGGACAAATCTGAATCAACC-3′)/CMV-CP-R (5′-TCAGA CTGGGAGCACCCC-3′) (Hu et al., 2016), respectively, via one-step reverse transcription polymerase chain reaction (RT-PCR) procedures. Assays were performed in total volumes of 25 μL cocktails containing 5× GoTaq^®^ Flexi buffer (Promega, Madison, WI, United States), 0.2 mM dNTPs, 1.5 mM MgCl_2_, 10 μM primers, 0.3 U of *Taq* polymerase (New England Biolabs, United States), 0.3 U of MMLV reverse transcriptase (New England Biolabs, United States), and sterile distilled water to make the final volume. Cycling conditions were an initial denaturation at 92°C for 3 min followed by 35 cycles at 95°C for 1 min, annealing at 60°C (RNA1) and 53°C (CP) for 1 min and extension at 2°C for 1.5 min with final elongation at 72°C for 7 min. Amplicons were analyzed on a 1.5% agarose gel stained with GelRedSafe (Promega, Madison, WI, United States) at 100 V for 1 h.

### Sequencing and Sequence Analyses

Products from both RT-PCR amplifications were ethanol-purified from selected CMV-positive samples and bidirectionally sequenced at Inqaba Biotec Company (South Africa). Nucleotides from both strands per amplicon were assembled and manually edited using BioEdit v.7.0.5 (Hall, 1999). Consensus sequences were obtained for each isolate and amplicon, verified by the nucleotide option of the basic local alignment search tool (BLASTn) (Altschul et al., 1990) and submitted to the GenBank database. Sequences of CMV RNA1 and CP genes from representative strains were downloaded from the National Centre for Biotechnology Information^1^ and used for subsequent analyses (Supplementary Table S1). Our sequences were compared with existing CMV isolates available at GenBank, while percentage similarities within the Nigerian population and those from the database were calculated using SDT v1.2 (Muhire et al., 2014) with pairwise gap deletions. Deduced proteins from both partial RNA1 and complete CP genes were also compared, and identities were obtained.

### Phylogeny and Recombination Analyses

Multiple alignments of nucleic acid and deduced amino acid sequences for both gene sets were separately performed on all CMV isolates used in this study (Supplementary Table S1) using ClustalW (Thompson et al., 1994) in BioEdit. The RNA1 and CP genes from peanut stunt virus strain P (accession numbers EU570236 and EU570238) were obtained as outgroups and included in the phylogenetic analyses. The base substitution models used for each gene set were selected using the jModelTest program (Darriba et al., 2012), supported by the corrected Akaike information criterion (AICc) values. Subsequently, the Tamura 3-parameter model, including heterogeneity of substitution rates among sites modeled using a discrete gamma distribution (T92 + G), was implemented for the RNA1 dataset, while the Kimura 2-parameter model, with the gamma-invariable mixture model (K2 + G + I), was adopted for the CP gene sequences. Bayesian phylogenetic trees were obtained using MrBayes v3.2.7 (Ronquist et al., 2012), with each analysis obtained from Monte Carlo Markov chain (MCMC) parameters set at ten million generations and sampled every 1,000 generations. Two independent runs were set per analysis with the minimum probability set at 0.05. Prior to convergence, the relative burn-ins were implemented at 25% for diagnostics, and trees generated were discarded. Chains were heated to 0.10, and trees were viewed using FigTree v1.4.4 (Rambaut, 2019).

Searches were conducted for recombination signatures within the partial RNA1 and complete CP gene populations using genetic algorithm recombination detection (GARD) and single breakpoint scanning (SBP) indices (Kosakovsky-Pond et al., 2006) as implemented by Datamonkey software (Weaver et al., 2018) at http://www.datamonkey.org. Evidence for the occurrence of recombination was further examined with algorithms within RDP v 4.13 (Martin et al., 2015) using the default settings and a Bonferroni corrected *P*-value cut-off of 0.05. Signals were confirmed true when detected by at least four detection methods with strong significance levels (*P* ≤ 0.05), in addition to the occurrence of putative parents across different phylogenetic clusters in test trees.

### Estimation of Genetic Diversity, Population Genetics, and Selection Pressure

The isolates obtained from this study, together with other Nigerian CMV RNA1 and CP gene sequences from the database (as of July 1, 2021), were used to infer population genetic structures of CMV in Nigeria. The estimated parameters comprised statistics such as nucleotide polymorphism (π), number of variable sites (*S*), haplotype diversity (Hd), number of haplotypes (*h*), number of mutations (Eta), statistic θ from *S* (θ-W) and the mean number of nucleotide differences between sequences (*k*). The deviation of the Nigerian CMV populations from neutrality for both gene datasets was determined via Tajima’s *D*, Fu and Li’s *D*, and Fu and Li’s *F* statistics (Tajima, 1989). Negative values indicate the occurrence of redundant low-frequency polymorphisms within the population prompted by population expansions, genetic hitchhiking or background selection, while positive values suggest nominal levels of low- and high-frequency polymorphisms that show balancing selection and/or a decrease in population size (Alabi et al., 2011). All genetic variability calculations and tests of neutrality parameters were obtained using DnaSP v5.10.01 software (Librado and Rozas, 2009).

The existence of selection pressures within the sequence populations was obtained using the single-likelihood ancestor counting (SLAC) method (Kosakovsky-Pond and Frost, 2005) as implemented in the HyPhy package (Kosakovsky-Pond et al., 2020) domiciled in Datamonkey software (Weaver et al., 2018). The status of selection pressures was obtained by calculating the ratio of the mean number of nucleotide differences between sequences per non-synonymous site (*d*_N_) and the mean number of nucleotide differences between sequences per synonymous site (*d*_S_). Selection pressure within the sequence populations was considered purifying when *d*_N_/*d*_S_ < 1, neutral when *d*_N_/*d*_*S*_ = 1 and diversifying when *d*_N_/*d*_S_ > 1 (Yang et al., 2000).

## Results

### Natural Occurrence of Cucumber Mosaic Virus Across Three Host Plants in Nigeria

A total of 106 samples were collected from 16 locations (Figure 1), out of which 24 were CMV-positive (Table 1). The leaf samples obtained from tomato, pepper and watermelon tested positive for CMV via RT-PCR, although infection was only obtained from eight locations in Oyo and Kwara states. Samples from Edo, Enugu and Akwa Ibom all tested negative for CMV, and there was no amplification from all African garden eggplant samples obtained across the areas sampled. Twelve positive isolates were selected for genomic sequencing of the partial CMV RNA1 segment, out of which 7 were further sequenced for the complete CP gene.

**TABLE 1:** Summary of leaf samples obtained from three host plants and tested for the presence of cucumber mosaic virus in Nigeria.

State	Location	Number of leaf samples (number of RT-PCR positives^a^)				
		*Solanum lycopersicum*	*Capsicum annuum*	*Solanum aethiopicum*	*Citrullus lanatus*	Total
Oyo	Ofamefa	2 (0)	2 (0)	0 (0)	0 (0)	4 (0)
Oyo	Ibapon	0 (0)	4 (2)	0 (0)	0 (0)	4 (2)
Oyo	Owolaake	3 (1)	4 (4)	2 (0)	0 (0)	9 (5)
Oyo	Imini	3 (0)	3 (0)	3 (0)	0 (0)	9 (0)
Oyo	Eruwa	3 (0)	3 (0)	2 (0)	2 (0)	10 (0)
Oyo	Ibidapo	3 (1)	2 (2)	2 (0)	0 (0)	7 (3)
Oyo	Igbo-Ora	2 (0)	2 (0)	0 (0)	2 (0)	6 (0)
Kwara	Lafiagi-1	2 (0)	2 (0)	0 (0)	3 (0)	7 (0)
Kwara	University of Ilorin	3 (1)	2 (1)	0 (0)	2 (2)	7 (4)
Kwara	Eyenkorin	2 (0)	2 (1)	0 (0)	2 (1)	6 (2)
Kwara	Ajase-Ipo2	3 (1)	1 (1)	0 (0)	1 (1)	5 (3)
Kwara	Olomu	1 (0)	2 (1)	2 (0)	3 (1)	8 (2)
Kwara	Lafiagi-2	3 (0)	3 (0)	0 (0)	3 (3)	9 (3)
Edo	Agenebode	2 (0)	1 (0)	2 (0)	0 (0)	5 (0)
Enugu	Nenwe	2 (0)	0 (0)	2 (0)	0 (0)	4 (0)
Akwa Ibom	Uyo	3 (0)	3 (0)	0 (0)	0 (0)	6 (0)
Total		37 (4)	36 (12)	15 (0)	18 (8)	106 (24)

*^a^Positive samples were confirmed using primers targeting the cucumber mosaic virus RNA1 gene as described by Wylie et al. (1993).*

### Presence of Subgroup IB Strains in the Nigerian Cucumber Mosaic Virus Population and Their Genetic Diversity

Sequencing of the partial RNA1 amplicons obtained from 12 RT-PCR-positive samples produced genomic fragments with nucleotides ranging from 506 to 543 bp and proteins with 144 amino acids (Table 2). These were deposited in the GenBank database under accession numbers MH798804-MH798810 and MW655573-MW655577. For the seven complete CP gene fragments, products ranged from 667 to 669 nucleotides with protein lengths between 217 and 218 aa (Table 2). The nucleotide sequences were deposited under the accession numbers OK107526-OK107532.

**TABLE 2:** Summary of partial RNA1 and RNA3 sequence characteristics and BLASTn results obtained from CMV isolates in this study.

Segment	Virus isolate	Host	Accession number	Number of nucleotides	Number of translated amino acids	Highest BLASTn similarity (%)	*E*-value	Alignment score	Highest query coverage (%)
RNA1	KW-OL-01	Watermelon	MW655573	543	144	97.79	0.0	≥200	99
RNA1	KW-AJ-02	Watermelon	MW655574	543	144	97.79	0.0	≥200	98
RNA1	KW-UN-03	Watermelon	MW655575	543	144	97.61	0.0	≥200	100
RNA1	KW-LF-04	Watermelon	MW655576	529	144	99.20	0.0	≥200	100
RNA1	KW-EY-05	Watermelon	MW655577	529	144	99.20	0.0	≥200	100
RNA1	10P_Ow1	Pepper	MH798804	509	144	96.84	0.0	≥200	99
RNA1	11P_Ow2	Pepper	MH798805	511	144	96.83	0.0	≥200	98
RNA1	15T_Ow3	Tomato	MH798806	509	144	96.84	0.0	≥200	99
RNA1	21P_Ibp	Pepper	MH798807	506	144	97.63	0.0	≥200	100
RNA1	22P_Ibp	Pepper	MH798808	508	144	97.63	0.0	≥200	99
RNA1	26P_Ibi	Pepper	MH798809	515	144	99.01	0.0	≥200	98
RNA1	29T_Ibi	Tomato	MH798810	507	144	92.90	0.0	≥200	100
RNA3	10P_Ow1	Pepper	OK107526	669	217	95.81	0.0	≥200	100
RNA3	11P_Ow2	Pepper	OK107527	668	217	94.90	0.0	≥200	99
RNA3	15T_Ow3	Tomato	OK107528	667	218	95.65	0.0	≥200	100
RNA3	21P_Ibp	Pepper	OK107529	669	217	95.07	0.0	≥200	100
RNA3	22P_Ibp	Pepper	OK107530	668	217	95.36	0.0	≥200	100
RNA3	26P_Ibi	Pepper	OK107531	667	217	96.10	0.0	≥200	100
RNA3	29T_Ibi	Tomato	OK107532	668	218	96.92	0.0	≥200	100

**FIGURE 3:**
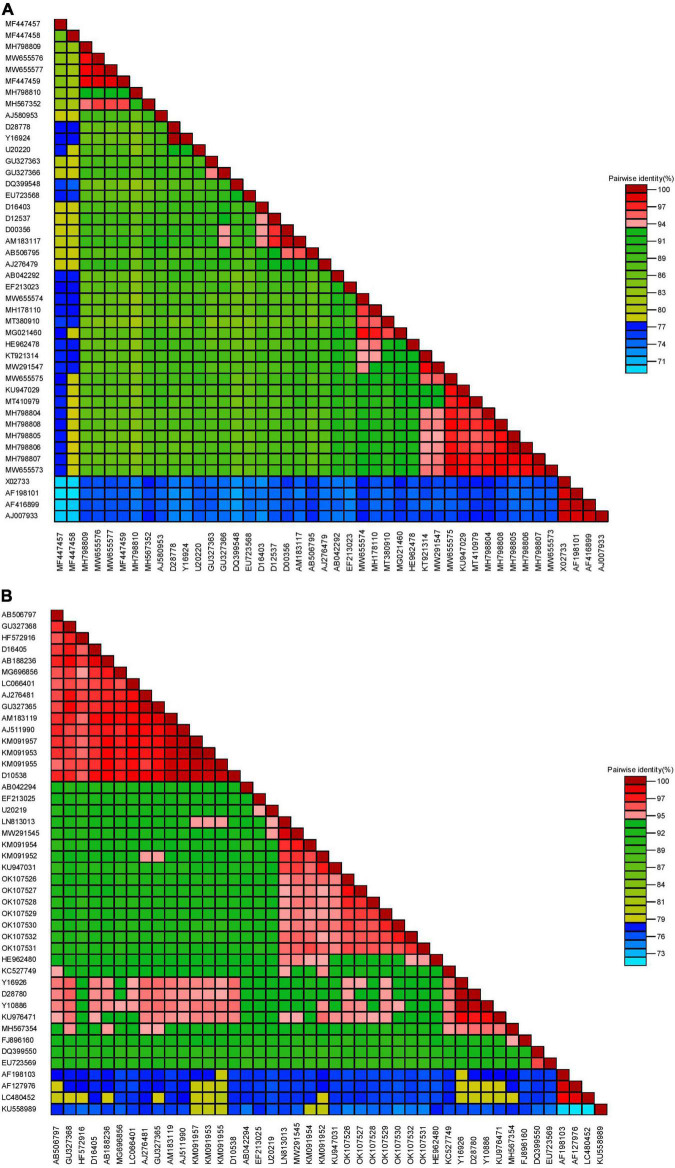
Pairwise similarities of partial RNA1 **(A)** and complete coat protein **(B)** genomes from CMV in Nigeria with selected worldwide sequences (see Supplementary Table S1 for details of isolates used for analyses).

BLASTn searches of the partial RNA1 genes revealed 92.90–99.20% nucleotide identities and 98–100% query coverage with CMV sequences present in GenBank (Table 2). These isolates shared 82.94–99.80% nucleotide and 85.42–100% amino acid identities with each other (Table 3) and 73.17–97.79% nucleotide sequence similarities with other CMV isolates from all subgroups worldwide (Figure 3A). Similar BLASTn searches using the complete CP gene populations produced 94.90–96.92% sequence identities with GenBank isolates, showing 98–100% query coverage (Table 2). The partial RNA3 nucleotides and the translated CP were 95.79–97.90% and 98.62–100% similar (Table 4) and shared 75.72–95.56% nucleotide identities with complete CP genes from global CMV isolates (Figure 3B). The translated proteins from both gene sets showed peculiar properties when aligned with other CMV subgroup I isolates, with nine unique amino acid changes observed within the RNA1 population (Supplementary Figure S1) and four amino acid alterations within the CP gene (Supplementary Figure S2).

**TABLE 3:** Percentage identity of RNA1a CMV isolates from natural host plants in Nigeria.

	MW655573	MW655574	MW655575	MW655576	MW655577	MH798804	MH798805	MH798806	MH798807	MH798808	MH798809	MH798810
MW655573	–	98.61	97.92	91.67	91.67	98.61	98.61	98.61	99.31	100.0	92.36	86.81
MW655574	93.55	–	96.53	93.06	93.06	98.61	97.22	97.22	97.92	98.61	93.75	88.19
MW655575	98.71	93.74	–	93.75	92.36	97.92	96.53	96.53	97.22	97.92	93.06	87.50
MW655576	87.64	86.12	88.21	–	98.61	93.06	90.28	90.28	90.97	91.67	99.31	92.36
MW655577	87.45	86.31	87.64	98.89	–	93.06	90.28	90.28	92.36	91.67	99.31	93.75
MH798804	97.64	91.94	97.45	86.61	86.42	–	97.22	97.22	97.92	98.61	93.75	88.19
MH798805	97.46	92.37	97.26	86.27	85.88	96.46	–	100.0	97.92	98.61	90.97	85.42
MH798806	97.84	92.73	97.64	86.42	86.02	96.46	99.80	–	97.92	98.61	90.97	85.42
MH798807	98.81	93.08	98.62	86.53	86.73	97.63	98.22	98.42	–	99.31	91.67	87.50
MH798808	98.23	92.52	98.03	86.79	86.19	97.83	97.64	97.83	98.42	–	92.36	86.81
MH798809	86.96	85.60	87.16	97.67	97.86	86.61	86.47	86.42	86.73	87.18	–	93.06
MH798810	84.52	82.94	84.72	92.86	93.06	84.52	83.53	83.53	84.13	84.13	93.65	–

*Nucleotide sequences are below the diagonal, while amino acid sequences are above the diagonal.*

**TABLE 4:** Percentage identity of RNA3 CMV isolates from natural host plants in Nigeria.

	OK107526	OK107527	OK107528	OK107529	OK107530	OK107531	OK107532
OK107526	–	99.54	99.08	99.54	99.54	99.08	99.54
OK107527	97.60	–	99.54	99.08	99.08	99.08	99.08
OK107528	97.14	96.99	–	98.62	98.62	99.54	98.63
OK107529	96.26	96.56	96.69	–	100.0	98.62	100.0
OK107530	96.71	96.10	96.09	97.00	–	98.62	100.0
OK107531	96.25	96.10	96.09	95.80	96.09	–	98.62
OK107532	95.79	96.10	95.95	96.40	97.29	9685	–

*Nucleotide sequences are below the diagonal, while amino acid sequences are above the diagonal.*

Further analyses of the RNA1 nucleotides showed 82.34–97.79% similarity with CMV isolates in subgroup IB and 82.63–90.27% identity with strains in subgroup IA (Supplementary Table S2). Deduced amino acids also revealed similar patterns, with 86.81–99.31% similarities with those from subgroup IB and 85.42–98.81% identities with isolates from subgroup IA (Supplementary Table S2). The isolates shared only 73.17–77.90% nucleotide and 79.02–86.11% amino acid similarities with CMV subgroup II isolates. Similarly, for the CP gene sequences, our nucleotides shared 89.37–93.42%, 92.96–96.92%, and 75.72–76.92% similarities with CMV isolates from subgroups IA, IB and II, respectively (Supplementary Table S3). The complete CP from the Nigerian CMV population shared 93.54–98.25% identity with isolates from subgroup IA, 95.85–100% identity with subgroup IB isolates and 81.94–83.79% identity with subgroup II isolates (Supplementary Table S3).

### Phylogenetic Relationships of Nigerian Cucumber Mosaic Virus Isolates

**FIGURE 4:**
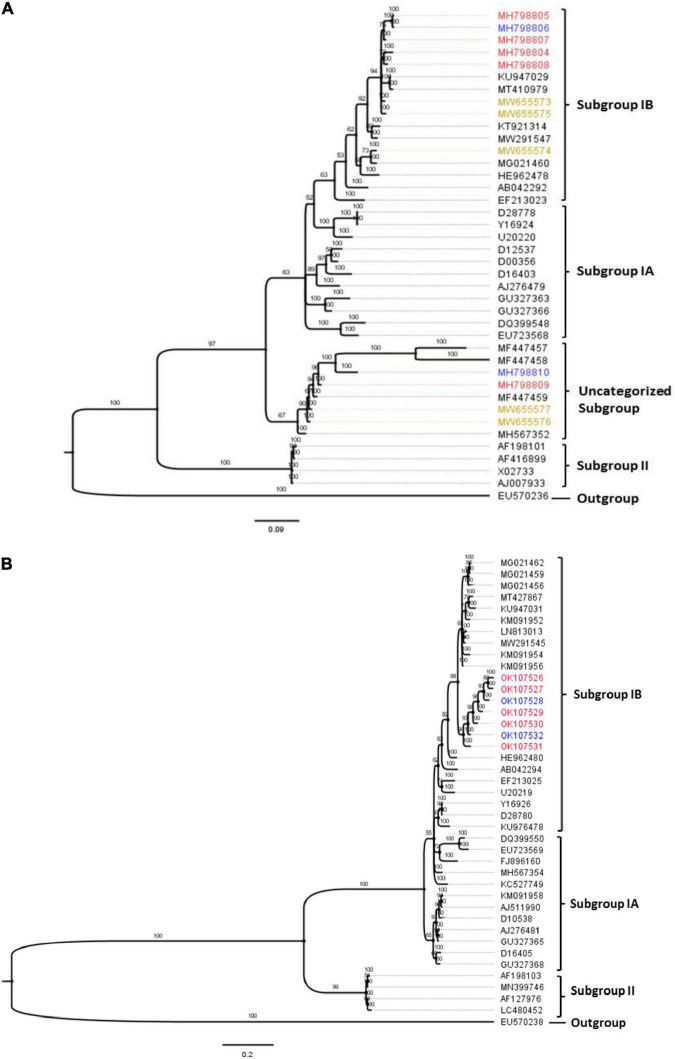
Bayesian phylogenetic analyses of CMV obtained from three hosts in Nigeria, with selected reference worldwide CMV strains. **(A)** Partial RNA1 genome. **(B)** Complete CP gene fragment. The trees were rooted using an isolate of peanut stunt virus strain P (accession numbers EU570236 and EU570238 for RNA1 and CP genes, respectively). The numbers above tree branches represent Bayesian inference posterior probability. Sequences obtained from this study were from tomato (blue), pepper (red) and watermelon (brown).

Phylogenetic analyses obtained via Bayesian probabilistic inferences categorized the CMV sequences in this study into two groups. First, within the RNA1 population, eight isolates clustered with other subgroup IB strains irrespective of their locations of origin or host. An isolate, ‘KW-AJ-02’ (MW655554), from watermelon formed a close relationship with an Ugandan CMV sequence (MG021460), as shown in Figure 4A. However, four isolates from this study formed a unique divergent subgroup with well-supported probability values. Isolates ‘26P_Ibi’ from pepper (MH798809), ‘29T_Ibi’ from tomato (MH798810), ‘KW-LF-04’ (MW655576) and ‘KW-EY-05’ (MW655577), both from watermelon, formed a distinctly separate subgroup with previously characterized CMV isolates from Nigeria (MF447457, MF447457, MF447459) and Kenya (MH567352). Likewise, all the CP gene sequences from the isolates also clustered with the subgroup IB strains, and this separation into a single clade was well supported (Figure 4B).

### Identification of a Putative Recombinant Within the Nigerian Cucumber Mosaic Virus Isolates

**FIGURE 5:**
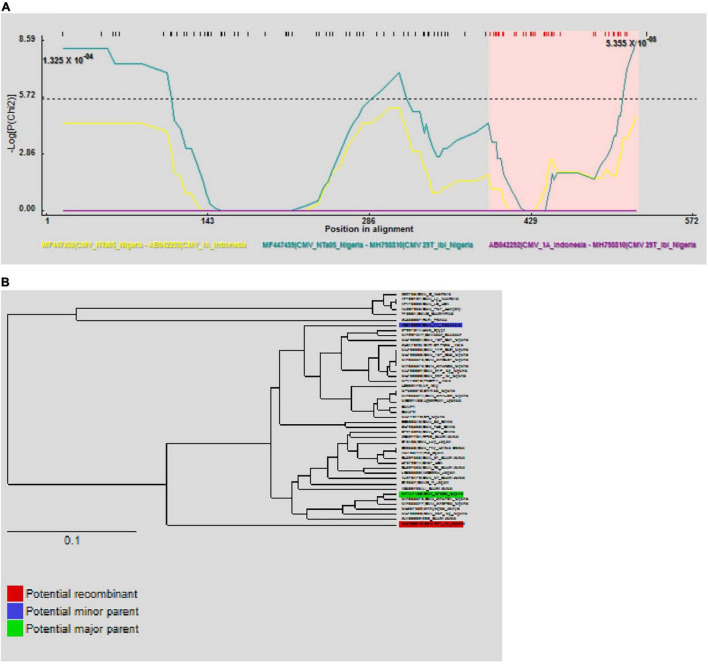
Putative recombination events on partial RNA1 sequences of CMV isolate ‘29T_Ibi’ on the RPD program. The –Log*P* maxChi values between the possible recombinant and its putative parents are shown and the pink colored region represents the potential region of recombination **(A)**. Phylogenetic inferences show putative parents in different clusters **(B)**.

The automated SBP and GARD tools in Datamonkey identified recombination signals within the CMV RNA1 sequences obtained in this study (data not shown). Further analyses using the algorithms implemented in the RDP4 software identified an isolate from tomato in Oyo State, ‘29T_Ibi’ (MH798810), as a putative recombinant (*P* ≤ 0.05), with subgroup IB isolates ‘N-Ta05’ (MF447459) and ‘1A’ (AB042292) as putative major and minor parents, respectively (Table 5). This was supported by recombination signals (Figure 5A) and phylogenetic reconstructions (Figure 5B) that categorized putative parents of the potential recombinant in separate clusters. No recombination signal was detected within the complete CP gene regions of the Nigerian CMV isolates.

**TABLE 5:** Recombination signals within the partial RNA1 sequences of CMV isolate ‘29T_Ibi’ (accession number MH798810) from Nigeria, as identified in RDP4.

Region		Parental sequences		*P*-values in RDP programs ^1^
Beginning	End	Major	Minor	
391	523	MF447459 (N-Ta05)	AB042292 (1A)	CHIMAERA (5.22E-03), MAXCHI (5.35E-05), SISCAN (5.12E-12), 3SEQ (1.50E-03)

*^1^P ≤ 0.05.*

### Population Genetic Structure and Selection Pressure Within Cucumber Mosaic Virus Populations in Nigeria

All CMV populations originating from Nigeria (partial RNA1 and RNA3 genes), obtained via our present study and those existing within the GenBank database, were pooled and analyzed. The genetic diversity within the CMV RNA1 gene populations from Nigeria was highest compared to the worldwide isolates. First, the nucleotide diversity (π) value of 0.12109 was highest in isolates from Nigeria (*n* = 17), compared to 0.09345 from other subgroup IB isolates (*n* = 17) and 0.08126 in subgroup IA sequences (*n* = 17) (Table 6). Similarly, ‘Eta’ and ‘*S*’ values showed the highest values in the populations from Nigeria, at 234 and 171, respectively. However, haplotype number and haplotype diversity values within the Nigerian population were similar to both subgroups IB and II populations. The nucleotide polymorphisms within the isolates, as revealed by Tajima’s *D*, Fu and Li’s *D*, and Fu and Li’s *F* statistics (Tajima, 1989), identified negative values for all the sequence datasets that did not statistically deviate from zero (*P* > 0.10) (Table 6). These results indicate the superfluous occurrence of low-rate polymorphisms within the RNA1 gene population, which may be caused by population proliferation, background selection or genetic hitchhiking. However, within the CP gene datasets, genetic diversity within the Nigerian population was comparable with subgroup IA and IB isolates. For example, the ‘π’ value within the Nigerian population (0.04804) was similar to the values obtained for subgroups IA (0.04550) and IB (0.04227) (Table 6). Conversely, a lower ‘*S*’ statistic was obtained from the Nigerian population, while ‘Eta’ values in subgroups IA and IB were almost twice the values recorded for the Nigerian sequences. The haplotype diversity within the Nigerian CP gene population was equivalent to the value obtained within subgroup II (Table 6). Additionally, Tajima’s *D* estimates were negative for the Nigerian CP isolates, while Fu and Li’s *D* and Fu and Li’s *F* statistics were positive, indicating population subdivision or bottlenecks. Collectively, these results confirm genetic diversity within the Nigerian CMV populations, with contrasting dynamics within the RNA1 and CP genes.

**TABLE 6:** Estimation of population genetic parameters for partial CMV RNA1 and RNA3 sequences from Nigeria in comparison with other worldwide strains.

Partial segment	Sequence population	*N* ^a^	*h* ^b^	*S* ^c^	Hd^d^	Eta^e^	π^f^	*k* ^g^	θ-W^h^	θ-Eta^i^	Tajima’s *D*	Fu and Li’s *D*	Fu and Li’s *F*
RNA1	Nigerian population (*n* = 17)	556	16	171	0.993	234	0.12109	58.3676	0.14360	69.2158	−0.6743	−0.6344	−0.7494
RNA1	Representative worldwide isolates: subgroup IB (*n* = 17)	557	16	159	0.993	194	0.09345	51.0250	0.10708	58.4649	−0.5774	−0.5210	−0.6226
RNA1	Representative worldwide isolates: subgroup IA (*n* = 17)	554	17	148	1.000	166	0.08126	43.9632	0.09076	49.1018	−0.4487	−0.1288	−0.2571
RNA1	Representative worldwide isolates: subgroup II (*n* = 17)	570	16	37	0.993	42	0.01225	6.9338	0.02195	12.4234	−1.8312	−1.8738	−2.1556
RNA3	Nigerian population (*n* = 12)	657	12	41	1.000	51	0.04804	16.6212	0.04881	16.8881	−0.0726	0.1266	0.0846
RNA3	Representative worldwide isolates: subgroup IB (*n* = 12)	657	11	77	0.982	82	0.04550	29.8909	0.04261	27.9962	0.3233	0.0965	0.1758
RNA3	Representative worldwide isolates: subgroup IA (*n* = 12)	657	11	83	0.985	85	0.04227	27.7727	0.04284	28.1468	−0.0618	−0.3410	−0.3051
RNA3	Representative worldwide isolates: subgroup II (*n* = 12)	657	12	35	1.000	35	0.01141	7.4848	0.01767	11.5899	−1.6047	−1.9569	−2.1246

*^a^Number of nucleotide sites; ^b^haplotype number; ^c^total number of segregation sites;^ d^haplotype diversity;^ e^total number of mutations; ^f^nucleotide diversity;^ g^mean number of nucleotide differences between sequences; ^h^Waterson’s estimate of population mutation rate based on the total number of segregating sites; ^i^Waterson’s estimate of population mutation rate based on the total number of mutations.*

The estimation of selection pressure occurring within CMV in Nigeria were determined via the ratios of non-synonymous substitution per non-synonymous sites (*d*_N_) and synonymous substitutions per synonymous sites (*d*_*S*_). Across all the CMV subgroups, *d*_N_/*d*_S_ ratios were less than 1 across datasets from both genes, indicating the occurrence of purifying or negative selection. Although subgroups IA and IB from isolates worldwide recorded similar numbers of sites and *d*_N_/*d*_S_ ratios within the partial RNA1 genomes, isolates from Nigeria and subgroup II recorded *d*_N_/*d*_S_ ratios of 0.293 and 0.254, respectively, at a *p*-value threshold of 0.1 (Table 7). The Nigerian CMV population had a *d*_N_/*d*_S_ ratio that was more than thrice that of other subgroup IB populations, with similar proportions also recorded for the number of sites for negative selection. However, the *d*_N_/*d*_S_ ratio within the Nigerian CP gene population was approximately half the values for subgroups IA and IB, albeit with almost five times the number of negative selections when compared with other isolates from subgroup IB (Table 7).

**TABLE 7:** Occurrence of selection pressure within the Nigerian CMV population compared with other CMV isolates worldwide.

Partial segment	Sequence population	Total number of sites	*log L*	*d*_N_	*d*_S_	*d*_N_/*d*_S_	Number of sites^a^	
							Positive selection	Negative selection
RNA1	Nigerian population (*n* = 17)	146	−1826.92	0.3482	1.1862	0.293	0	13
RNA1	Representative subgroup IB worldwide isolates (*n* = 17)	146	−1948.69	0.1479	1.7015	0.087	0	36
RNA1	Representative subgroup IA worldwide isolates (*n* = 17)	146	−1833.03	0.1351	1.5066	0.089	0	33
RNA1	Representative subgroup II worldwide isolates (*n* = 17)	146	−873.37	0.0441	0.1732	0.254	0	3
RNA3	Nigerian population (*n* = 12)	218	−1860.12	0.0444	0.7793	0.057	0	24
RNA3	Representative subgroup IB worldwide isolates (*n* = 12)	218	−1481.26	0.0403	0.3955	0.102	0	5
RNA3	Representative subgroup IA worldwide isolates (*n* = 12)	218	−1554.65	0.0583	0.4119	0.141	0	8
RNA3	Representative subgroup II worldwide isolates (*n* = 12)	218	−1168.36	0.0315	0.1218	0.258	0	1

*^a^Selected at p-value threshold of 0.1.*

## Discussion

Cucumber mosaic virus, a ubiquitous virus occurring in hundreds of host plants worldwide, causes huge losses to economic plants in terms of quality and quantity. Here, we have identified highly diverse CMV populations in solanaceous crops (pepper and tomato) and cucurbit (watermelon) occurring in Nigeria, together with their partial genetic properties. Although only partial sequences of the RNA1 and RNA3 segments were obtained, the polymorphisms identified as well as analyses of population genetics and selection pressure are sufficient to present a highly diverse, natural CMV population in Nigeria. First, within the hosts sampled, the occurrence of multiple virus infections cannot be totally overruled, especially within fruit and leafy vegetables in Nigeria, where other viruses have also been identified (Alegbejo, 2015; Arogundade et al., 2015; Fakolujo, 2018; Adediji, 2019; Ekpiken et al., 2021). Among the hosts sampled, only very mild mosaics were observed on African garden eggplants, and no CMV was amplified. This is in agreement with the findings of Al-Ali et al. (2013), who observed little or no virus symptoms associated with African garden eggplants. However, this study affirms the increasing host range of CMV infection in Nigeria. Although previous studies from Nigeria (Salem et al., 2010; Kayode et al., 2014; Adediji, 2019; Ekpiken et al., 2021) have obtained some CMV sequences, this is the first comprehensive description of its population genetics across multiple natural hosts in the country. This is important, as diversity studies of CMV populations provide insights into their possible evolutionary trend (Koundal et al., 2011).

Sequence analyses using both RNA1 and RNA3 gene sequences obtained in this study clearly identified subgroup IB as the major CMV strain present in the country. We previously identified CMV from plants in Nigeria that belong to subgroup IB (Adediji, 2019), although the occurrence of subgroup IA has also been reported (Kayode et al., 2014). Thus, the presence of more CMV isolates belonging to subgroup IB has now been confirmed in Nigeria and appears to be more prevalent, especially across different agro-ecologies and hosts. The strain is reported to have Asian origins (Roossinck, 2002; Hasanvand and Shams-Bakhsh, 2017), and its expansion into Africa points to the looming threat the virus poses to food security, especially for the continent’s most populous country. CMV isolates belonging to subgroup IB have been found to occur in East Africa (Kidanemariam et al., 2019), and this report confirms its expansion into West Africa and probably beyond. However, this study established high diversity within the subgroup IB populations in Nigeria. Similar diversity patterns within the RNA1 and CP gene segments of subgroup IB populations have also been previously reported (Revathy and Bhat, 2017; Giakountis et al., 2018; Pavithra et al., 2019). Intriguingly, diversity was high across both evaluated genes even when compared with existing Nigerian CMV sequences in the database. Isolates from Nigeria were more similar to isolates from Uganda and India than others in Nigeria, suggesting transboundary interrelationships across the isolates.

Interestingly, the phylogeny revealed that the subgroup IB strains in this study formed distinct subclades, confirming the diversity within the isolates and suggesting that the Nigerian CMV populations circulating in host plants could be from at least two separate events of introduction. The RNA3 populations also clustered in subgroup IB, forming a unique subgroup within the representative global sequences and indicating a distinct lineage within the population. Additionally, our RNA1 isolates that clustered into the distinct, putatively novel subgroup had a close phylogenetic relationship with a CMV isolate from Kenya (Figure 4A). Indeed, the Kenyan isolate (accession number MH567352) has been identified as a reassortant strain of Asian origin (Mutuku et al., 2018). This shows that new CMV strains may be emerging and widespread across Africa within various hosts and might be driven by recombination. We have previously postulated that some CMV strains in Nigeria may be novel, as they do not follow the current delineated strain categorization (Adediji, 2019). However, only analysis of the complete virus RNA fragments will unravel the properties and behaviors of the virus, especially within multiple hosts across different sites in Nigeria. In addition, the CMV populations from Nigeria did not cluster on the basis of host or area of sampling, although locational segregation of CMV strains has been previously identified in California, United States (Lin et al., 2004).

Cucumber mosaic virus, like other RNA viruses, is known to have high mutation rates driven by recombination (Bonnet et al., 2005; Nouri et al., 2014; Hasiów-Jaroszewska et al., 2017; Ouedraogo et al., 2019), and our study identified a putative recombinant within the RNA1 population (isolate ‘29T_Ibi’ from tomato in Oyo State) occurring within the Nigerian population. The high levels of genetic diversity and strain emergence identified in this study may be driven by recombination and/or reassortment of the genomes. This was confirmed via phylogeny and suggests the presence of distinctly evolving strains that differ from currently recognized CMV categories. To our knowledge, this is the first report of a recombinant CMV subgroup IB isolate from West Africa. The presence and occurrence of such recombinants in numerous hosts and possible seasonal transmission via aphids (Kone et al., 2017) may further drive its spread and evolution in Nigeria and across the subcontinental region. No significant recombination signal was detected within the CP genes, suggesting evolutionary conservation within the genomic region. Several authors (Roossinck, 2002; Bonnet et al., 2005; Thompson and Tepfer, 2009; Nouri et al., 2014) have identified recombination within the CMV RNA3 segment which encompasses the CP gene and the 3′ untranslated region. Our study did not amplify the full genome of RNA3, hence the reason why recombination might have been undetected. Indeed, more significant evidence could be detected within CMV in Nigeria using full-length fragments of the virus RNAs, as similarly postulated by Pavithra et al. (2019), as well as having a more robust population across diverse host plants. There is a need to obtain more information on intra- and intergenomic recombination occurring in other coding and non-coding regions within the genomes, as well as reassortments within and across CMV strains. As the impact of climate change on agriculture and crop cultivation continues to unravel worldwide, the factors that drive genomic recombination in plant RNA viruses will have an intense impact on their properties, evolution and survival (Nagy, 2008).

Furthermore, estimates of population genetics reveal the presence of negative selection pressure within the CMV populations in these host plants. However, the Nigerian population was unique, as the frequency of purifying selection within the RNA1 sequences was less than half the rates within the general subgroup IB, while the CP gene fragments revealed very a high number of negative selections comparable to subgroups IA, IB and II. The occurrence of negative selection within the CMV CP gene has been previously reported (Liu et al., 2009; Nouri et al., 2014; Hasanvand and Shams-Bakhsh, 2017). However, the properties within the Nigerian population could be due to the widespread occurrence of the virus across several hosts, driven by multiple introductions and independent coevolution across various spatial and temporal scales. The *d*_*N*_/*d*_*S*_ values within the partial RNA1 sequences from Nigeria were similar to those of the global subgroup II isolates, while the values for the CP genes were considerably low, thus indicating varying evolutionary pressures within the genomes of the CMV population. Consequently, characterization of full CMV genomes from these isolates will provide more information and could make a stronger case for distinct CMV strains with unique properties occurring from Nigeria. Although partial genome fragments were investigated in this research, the occurrence of new CMV strains cannot still be ruled out. The virus is known to be cosmopolitan and can be subjected to several evolutionary interplays across its various hosts. However, definitive evidence for the occurrence of novel strains must be supported by ecological and epidemiological evidence and not rely only on genomic properties.

Conclusively, our study has established unique properties of CMV populations in Nigeria using only partial gene fragments. The complete genome sequence of virus RNA fragments will help to understand the unique properties of CMV populations circulating in the country, unraveling more insights into the dynamics of a widespread and cosmopolitan plant virus. Ultimately, this information is crucial for formulating robust detection and management schemes for the virus to ensure food security.

## Data Availability Statement

The datasets presented in this study can be found in online repositories. The names of the repository/repositories and accession number(s) can be found in the article/Supplementary Material.

## Author Contributions

AOA conceived the research. AOA, OSB, NBI, and GIA participated in the experimental design and coordination. OAA, AOA, TIF, JMA, and SM collected the field samples. OAA, AOA, TIF, and JMA performed the experiments. OAA, AOA, and JMA analyzed the data and prepared all figures. JMA, MAA, MMG, SM, FSIQ, and GEB provided technical assistance. AOA, OSB, and GIA supervised the research. AOA wrote the draft manuscript. OAA and AOA proofread and finalized the manuscript. All authors read and approved the final version.

## Conflict of Interest

The authors declare that the research was conducted in the absence of any commercial or financial relationships that could be construed as a potential conflict of interest.

## Publisher’s Note

All claims expressed in this article are solely those of the authors and do not necessarily represent those of their affiliated organizations, or those of the publisher, the editors and the reviewers. Any product that may be evaluated in this article, or claim that may be made by its manufacturer, is not guaranteed or endorsed by the publisher.

## Footnotes

1
http://www.ncbi.nlm.nih.gov
