# Supplementary material for: Genetic Structure of Cucumber Mosaic Virus From Natural Hosts in Nigeria Reveals High Diversity and Occurrence of Putative Novel Recombinant Strains
Source: Front Microbiol. 2022 Feb 10;13:753054. doi: 10.3389/fmicb.2022.753054 (PMC8866732; doi:10.3389/fmicb.2022.753054)
Supplement: Supplementary file 2 [file Table_2.DOCX]

**SUPPLEMENTARY FIGURE S2 |** Alignment of the *Cucumber mosaic virus* coat protein gene from three hosts in Nigeria with other CMV sequences across subgroup I from GenBank database showing unique amino acid site alterations (see sequences Supplementary Table S1 for details of isolates).
